# Molecular differentiation of five *Cinnamomum camphora* chemotypes using desorption atmospheric pressure chemical ionization mass spectrometry of raw leaves

**DOI:** 10.1038/srep46579

**Published:** 2017-04-20

**Authors:** Xiali Guo, Meng Cui, Min Deng, Xingxing Liu, Xueyong Huang, Xinglei Zhang, Liping Luo

**Affiliations:** 1 School of Life Sciences, Nanchang University, Nanchang, Jiangxi 330031, China; 2 State Key Laboratory of Food Science and Technology, Nanchang University, Nanchang, Jiangxi 330031, China; 3 Jiangxi Key Laboratory for Mass Spectrometry and Instrumentation, East China Institute of Technology, Nanchang, Jiangxi 330013, China

## Abstract

Five chemotypes, the isoborneol-type, camphora-type, cineole-type, linalool-type and borneol-type of *Cinnamomum camphora* (L.) Presl have been identified at the molecular level based on the multivariate analysis of mass spectral fingerprints recorded from a total of 750 raw leaf samples (*i.e.*, 150 leaves equally collected for each chemotype) using desorption atmospheric pressure chemical ionization mass spectrometry (DAPCI-MS). Both volatile and semi-volatile metabolites of the fresh leaves of *C. camphora* were simultaneously detected by DAPCI-MS without any sample pretreatment, reducing the analysis time from half a day using conventional methods (e.g., GC-MS) down to 30 s. The pattern recognition results obtained using principal component analysis (PCA) was cross-checked by cluster analysis (CA), showing that the difference visualized by the DAPCI-MS spectral fingerprints was validated with 100% accuracy. The study demonstrates that DAPCI-MS meets the challenging requirements for accurate differentiation of all the five chemotypes of *C. camphora* leaves, motivating more advanced application of DAPCI-MS in plant science and forestry studies.

Chemotypes are organisms categorized under the same species, subspecies or varieties, with differences in the composition of secondary metabolites^1^. Minor genetic and epigenetic changes with little or no effect on morphology or anatomy may produce large changes in the chemical phenotype. It is a widespread phenomenon that chemotypes are diverse in medicinal plants^2^,^3^,^4^,^5^,^6^,^7^. For example, the chemotypes which produce highly qualified essential oil products have been discovered in numerous species of plants^5^,^7^,^8^,^9^,^10^ including mint, elsholtzia, camphor, basil, houttuynia and patchouli etc. Therefore, classification of natural plants according to the chemotypes is essentially important for both industrial production and academic research.

*Cinnamomum camphora* (L.) Presl is native to the south of the Yangtze River in China, and has been introduced to many other countries^11^,^12^. *C. camphora* has long been prescribed in traditional Chinese herb medicines for the treatment of inflammation-related diseases such as rheumatism, sprains, bronchitis, asthma, indigestion, muscle pains and lasioderma serricorne^11^,^13^. Roots, stems, leaves and wood of *C. camphora* are all rich in essential oil which contains chemicals such as camphor, linalool, safrole and cineole as the major valuable ingredients^14^,^15^. Accordingly, *C. camphora* is of increasing importance as a source of essential oil in recent years, especially for the production of natural linalool which is still preferred over the synthetic form for fragrant applications^16^. Significant phytochemical variation has been found in the main constituents of essential oil originating from *C. camphora* trees of varied chemotypes such as the isoborneol-type, camphora-type, cineole-type, linalool-type and borneol-type^17^,^18^. However, there is no significant difference in morphology among all the five chemotypes. This makes it extremely difficult for accurately classifing *C. camphora* chemotypes with sensory methods accordingly.

In traditional processes, the typical extraction processes such as hydrodistillation, soxhlet, steam distillation, simultaneous distillation and extraction (SDE)^19^,^20^,^21^ are required before optical spectroscopy^22^, gas chromatography (GC)^23^, gas chromatography mass spectrometry (GC-MS)^19^ or Fourier transform infrared (FT-IR) spectroscopy^24^ can be employed to detect the volatile and phenolic compounds from plants prior to the recognition of their chemotypes. Clearly, adding pretreatment processes increases cost of the analytical time, consumption of the sample and uncertainty of the results.

Requiring no tedious sample pretreatment, desorption atmospheric pressure chemical ionization mass spectrometry (DAPCI-MS) has been used for the direct analysis of various samples without separating the complex matrices^25^,^26^,^27^. For example, the application of DAPCI-MS for trace detection of melamine in milk, eggs and kidney stone samples^28^,^29^,^30^, discovery of sinapine in radish root^31^, differentiation of tea products^32^, fruits of *Fructus schisandrae*^33^ and raw propolis^34^ have been documented in the literature with dramatically improved analytical throughput. In the previous work^12^, products such as combs made of materials containing no camphor chemicals were successfully differentiated from the genuine ones by DAPCI-MS, inspiring attempts to utilize DAPCI-MS for classification of *C.* camphora chemotypes with raw leaves. To the best of our knowledge, no similar studies have been reported on the direct discrimination of chemotypes of plants, including *C. camphora* by either DAPCI-MS or other techniques based on ambient mass spectrometry.

In this study, DAPCI-MS was used to rapidly fingerprint the chemotypes of *C. camphora* leaves. The principal component analysis (PCA) and cluster analysis (CA) of the DAPCI-MS spectral fingerprint data were carried out to draw the 3-dimensional (3D) score plots, which visualized the differentiation of the five chemotypes of *C. camphora* leaf samples.

## Results and Discussion

### Desorption atmospheric pressure chemical ionization mass spectrometry analysis of *C. camphora* leaves

**Figure 1:**
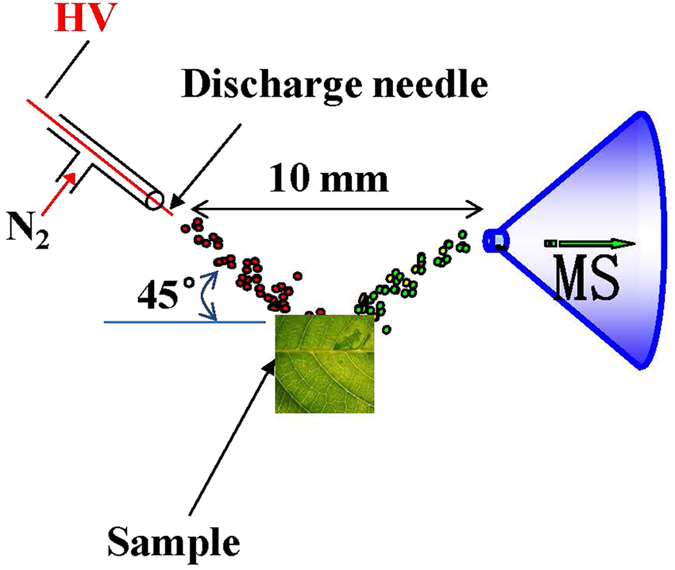
Schematic illustration of DAPCI setup for mass spectrometry analysis of raw fresh leaf samples.

In order to maintain a stable signal at the maximal level, the DAPCI working parameters (Fig. 1) were experimentally optimized as described in methods, including the distance and angle between the sample and the tip in DAPCI source, voltage, and the temperature. Under the optimized experimental conditions, the DAPCI mass spectra for all camphor fresh leaves were recorded. The acceptable stability and reproducibility were observed based on repeatable mass spectra of different samples. For example, for given signals at *m/z* 135, 137, and 153, the signal intensity levels were varied within a reasonably narrow range, giving relative standard deviation (RSD) values of 3.8∼6.2% for every 10 measurements. The RSD values were also found to be increased from 3.8∼6.2% to about 8∼12% for the same leaf sample but measured in 3 days, which was still reasonable for measuring the untreated raw leaf samples with DAPCI-MS. For a large amount of sample analysis, the analysis time for the whole sample set might last for several weeks. For such a long time, it could be helpful to correct the baseline drift or any other notable variation of the instruments using a standard amino acid solution (ca 10 ppb).

### Chemical profiling of *C. camphora* raw leaves by DAPCI-MS

**Figure 2:**
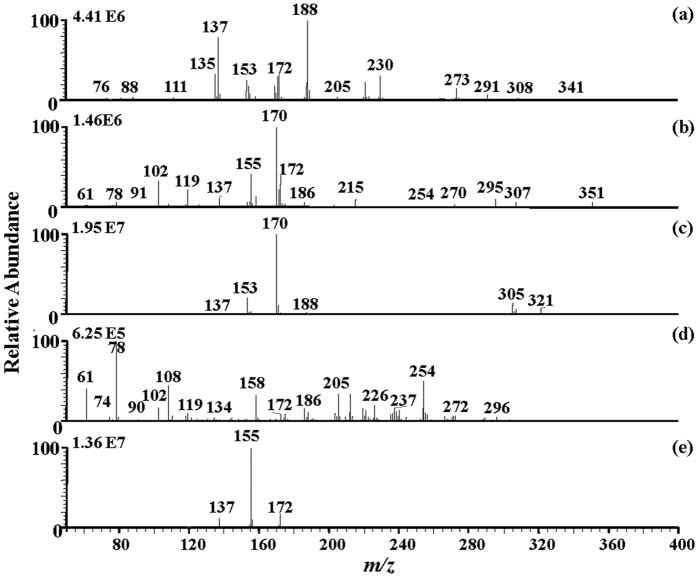
DAPCI-MS spectral fingerprints of fresh *C. camphora* leaves obtained from 5 chemotypes. (**a**) linalool-type; (**b**) borneol-type; (**c**) camphora-type; (**d**) isoborneol-type; (**e**) cineole-type.

The DAPCI-MS fingerprints of 5 chemotypes of *C. camphora* leaves were shown in Fig. 2. Obviously, the spectra of the linalool-type (Fig. 2a), isoborneol-type (Fig. 2d), cineole-type (Fig. 2e), and camphora-type (Fig. 2c) leaves showed quite a broad diversity, while the borneol-type (Fig. 2b) and camphora-type (Fig. 2c) showed high similarity in the chemical fingerprints. Cineole-type displayed the lowest number of signals (four major peaks). Isoborneol-type displayed the largest number of signals (18 major peaks). For the most spectra, the molecules of relatively low molecular weight were detected as the protonated molecules between *m/z* 100 and 280. For instance, the main peaks were *m/z* 135, 137, 153, 172, 188, 230 in the spectra of linalool-type leaves. In the spectrum of the borneol-type, the main peaks were *m/z* 170, 155, 172, 102, 119 while abundant peaks at *m/z* 170, 153 and small peaks at *m/z* 137, 188 *etc*. were detected in the camphora-type leaves. In the cineole-type spectral fingerprints, the signal intensity of the peak at *m/z* 155 was the strongest, reflecting that cineole (MW 154) naturally was of the highest content in the cineole-type essential oil^15^,^18^. It is widely known that the different medicinal properties of these 5 *C. camphora* chemotypes arise from the diversity of their chemical composition. In the results mentioned above, detailed chemical differences in their leaves were acquired with DAPCI-MS, providing an explicit statistic base for traditional usage of *C. camphora* chemotypes previously identified mainly by sensor methods with low precision and accuracy. On the other hand, major MS peaks can be selected as specific markers for the identification of each *C. camphora* chemotype. Given the high cost of MS analysis, selection of several characteristic compounds out of a large set of spectrum data as identification markers is obviously highly practical. To address this issue, our studies on *C. camphora* chemotypes using DAPCI-MS could provide a novel insight in terms of feasibility, efficiency and accuracy.

**Figure 3:**
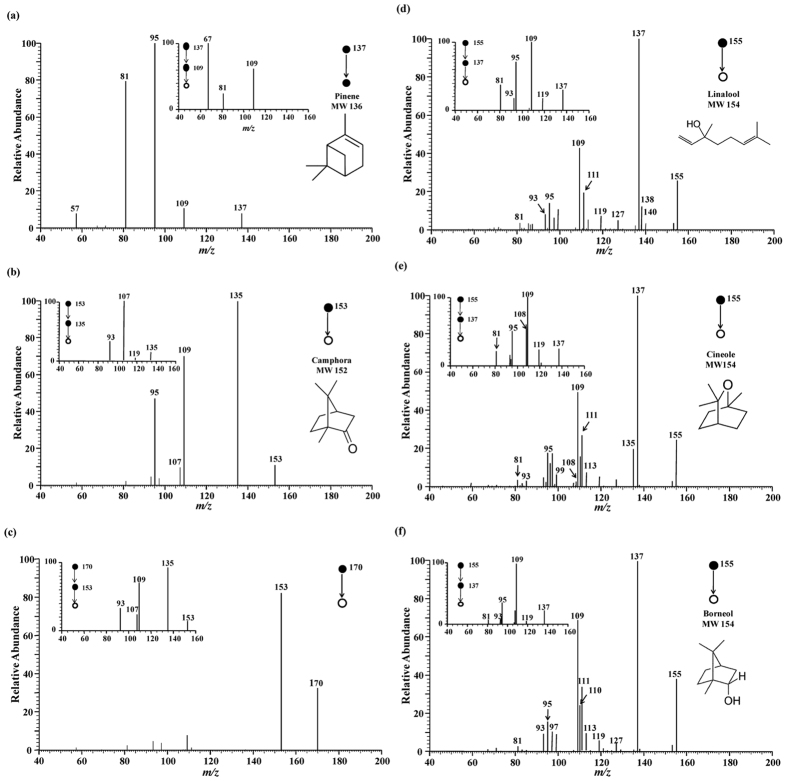
DAPCI tandem mass spectra of analyte ions of interest. DAPCI-MS^3^ spectrum of (**a**) pinene (*m/z* 137); (**b**) [M + NH_4_]^+^ (*m/z* 170); (**c**) camphor (*m/z* 153); (**d**) linalool (*m/z* 155); (**e**) cineole (*m/z* 155); (**f**) borneol (*m/z* 155).

The MS^2^ spectrum of the ions at *m/z* 137 was shown in Fig. 3a, which was tentatively assigned to protonated pinene. The major fragment ions at *m/z* 109, 95 and *m/z* 81 were generated from ions of *m/z* 137, probably by the loss of neutral fragments of C_2_H_4_, C_3_H_6_ and C_4_H_8_, respectively. Further fragmentation for the ions of *m/z* 109 produced the ionic fragments of *m/z* 95, 81 and 67, probably due to the loss of neutral fragments of CH_2_, C_2_H_4_, C_3_H_6_, respectively. These fragments fully matched the data obtained using a proton transfer reaction ion trap mass spectrometry by other groups^35^ and the data of authentic pinene compound using DAPCI-MS by our group (Figure S1), confirming the successful detection of pinene from the fresh leaf samples.

Camphor (MW152, *m/z* 153) was the highest content reaching more than 30% in the camphora-type essential oil, and up to 10% in the borneol-type. Under the open air corona discharge conditions, camphor molecule forms adducts with ambient ammonia easily. For example, the abundant peak at *m/z* 170 present from the camphora-type samples was identified as the protonated ammonia adduct with camphor [M + NH_4_]^+^. Upon the collision-induced dissociation (CID) process, the precursor ions of *m/z* 170 yielded the protonated camphor at *m/z* 153 (Fig. 3b). Such daughter ions (*m/z* 153) produced the characteristic fragmentation pattern (inset of Fig. 3b), which was identical to that generated by the protonated camphor (Fig. 3c). For example, as shown in Fig. 3c, the protonated camphor (*m/z* 153) produced major fragment ions at *m/z* 135, 109, and 95 by loss of H_2_O, C_2_H_4_O, and C_3_H_6_O, respectively. The fragmentation pattern was consistent with the results of electron impact (EI) ionization^12^,^36^. Furthermore, the fragmentation pattern has been validated using authentic compound of Camphora, and the fragmentation pattern of protonated camphora (*m/z* 153) was shown in Figure S2.

The 3 isomer compounds of linalool, cineole and borneol are the most characteristic volatile components^18^ in the linalool-type, cineole-type and borneol-type *C. camphora* leaves, respectively. In the positive ion mode, these compounds (MW 154) were detectable as the protonated molecules [M + H]^+^ at *m/z* 155. According to Shu *et al*.^37^, linalool generated major peaks at *m/z* 155 [M + H]^+^, and 137 [M + H-H_2_O]^+^, accompanied by small peaks at *m/z* 111, 109, and 95, by the loss of C_2_H_2_, C_2_H_4_, and C_3_H_6_ from the fragment of *m/z* 137, respectively. In this study, these ion peaks were also observed in the DAPCI-MS spectrum, as shown in Fig. 3d. These fragments were also detected from the authentic linalool, as shown in Figure S3, indicating the successful detection of linalool from the fresh leaves. Under the experimental conditions, the product ion spectrum of protonated cineole were similar to those observed in the linalool case (Fig. 3e), and the featured ionic fragments of cineole observed in MS^3^ spectrum were detected at *m/z* 81, 95, 108, 109 and 119 as shown in the inset of Fig. 3e. The major fragment ions were also detected with a similar pattern using authentic cineole (Figure S4). Similarly, the detection of borneol was also validated with authentic compound to match the CID fragments detected in the raw sample. Figure 3f showed the CID paths of the compound detected from the leaf sample, which matched the featured fragment ions of standard borneol as shown in Figure S5. These results were also in good agreement with those obtained with VAE-DLLME-GC-MS method^38^.

### Principle component analysis of leaves of 5 *C. camphora* chemotypes

**Figure 4:**
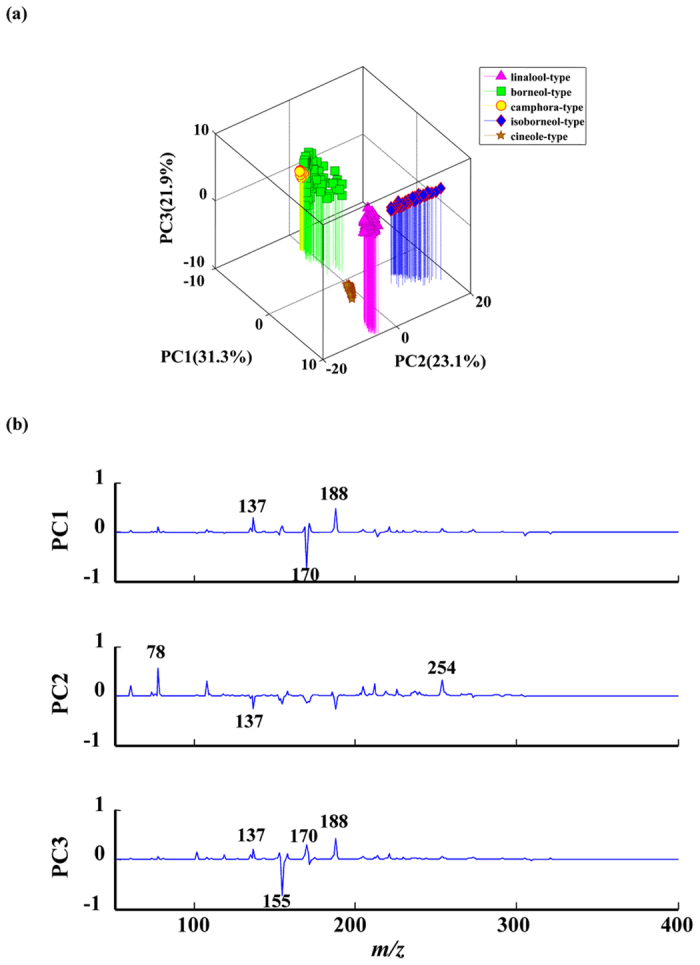
Principal component analysis results of DAPCI-MS data from leaves of 5 *C. camphora* chemotypes. (**a**) 3D plot of PCA score results; (**b**) PCA loading results for the PCs.

To visualize the molecular differences among the chemotypes of *C. camphora*, principal component analysis was performed for the DAPCI-MS spectral data with Matlab software. The 3D PCA score plot matrix was shown in Fig. 4. Three PCs (PC1 eigenvalue 31.3%, PC2 eigenvalue 23.1%, PC3 eigenvalue 21.9%) representing about 76.3% of the total variance, were selected for modeling. A total of 750 data points of samples from 5 *C. camphora* chemotypes were clustered tightly in the PCA plots (Fig. 4). However, the camphora-type and borneol-type occupied adjacent area, indicating that camphor leaves of the two chemotypes contained relatively similar volatile components. Furthermore, the blind samples of 5 leaves were tested using the same method, which indicated that all the 5 leaves belonged to the linalool-type. More interestingly, the GC-MS experiments performed on the 5 blind samples confirmed the results obtained using the method established here (data not shown), showing that DAPCI-MS combined with PCA could be used conveniently to differentiate *C. camphora* and could be a promising method to rapidly identify plant chemotypes.

### Cluster analysis of leaves of 5 *C. camphora* chemotypes

**Figure 5:**
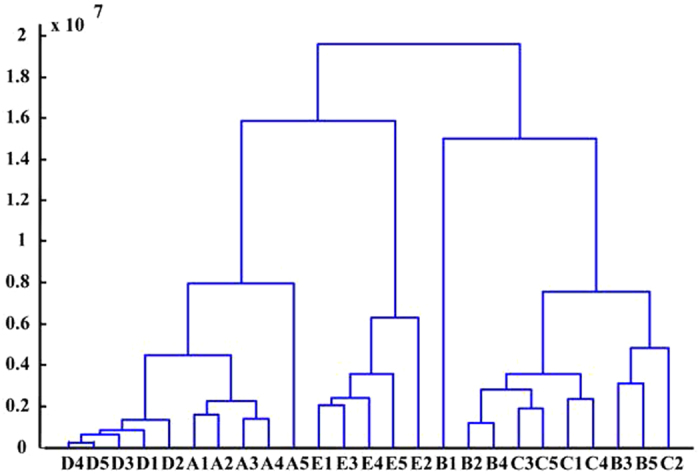
Dendrogram of the cluster analysis to validate the principal component analysis results. (A1-A5) linalool-type; (B1-B5) borneol-type; (C1-C5) camphora-type; (D1-D5) isoborneol-type; (E1-E5) cineole-type.

Cluster analysis (CA), an alternative data processing method to cluster abstract objects based on their similarities, was commonly used for classification of unknown samples^32^,^39^. In this study, CA was used to cross check the results of PCA for method validation since the results of CA are simple and intuitive^40^ in comparison with those obtained by PCA. Accordingly, a total of 750 DAPCI mass spectra recorded from 5 chemotypes of *C. camphora* samples were processed by CA using Matlab software. The Euclidean distance between each sample was 0–1.8 × 10^7^ and the aggregation process was expressed in the diagram as shown in Fig. 5, in which 5 categories of the isoborneol-type, borneol-type, linalool-type, cineole-type and camphora-type generally were clearly classified. These results were in good agreement with those of PCA. Both in PCA and CA, the distances between borneol-type and camphora-type were the closest thus the differentiation between them was compromised to a certain degree. This could possibly reflect the similarities in their chemical properties. This point was also supported by a former study on volatile chemical compositions in leaves of different *C. camphor*a chemotypes conducted with static headspace-gas chromatography-mass spectrometry (SHS-GC-MS) that combined with multivariate analyses revealed a very close analogy of 6 volatile components (12 in total selected for mapping) in these two chemotypes^18^.

The capability of DAPCI-MS for many types of application including screening camphor wood products^12^ has been investigated by our group in recent years. However, the previously published work^12^ focused on differentiation of camphor wood products from other types of materials containing no camphor-wood-related chemicals. The current study further developed the method of DAPCI-MS for classification of *C. camphora* chemotypes. As already partially presented in this paper, the major work of this study is quite different from the previously published one^12^. Technically speaking, the difficulty of the current work is also much more challenging than the previous work^12^. Therefore, the previous work brings no hurt to the current manuscript in any degree.

Requiring no dedicated sample pretreatment, DAPCI-MS is fast to profile the chemical fingerprints of samples. This feature makes it suitable for high throughput applications such as differentiation of plant chemotypes. However, limited by the mass analyzer used in this work, it was not able to resolve many compounds, resulting in difficulties in identification of certain types of analytes. In comparison with either GC-MS or LC-MS, the matrix effect still plays a significant role in ambient ionization mass spectrometry such as DAPCI-MS, although in many cases enough information has been confidently obtained by DAPCI-MS with molecular specificity. This study demonstrated that DAPCI-MS in combination with PCA and CA was a feasible technique for high-throughput differentiation of raw leaves from various chemotypes of *C. camphora*. The technique was in the ambient environment without any sample pretreatment. Compared to traditional techniques, this technique detected the plant *in stiu* without any extraction of volatile and phenolic compounds. For headspace extraction method developed in recent years^41^,^42^, half an hour at least is needed for heating and equilibration, without regard for the subsequent GC-MS analysis. Furthermore, under field work conditions, especially for medical plants such as camphor trees harboring diverse chemotypes with highly morphological similarity, rapid and accurate identification of each chemotype should be of great help to researchers. The rapid method established here could provide a theoretical basis for a portable apparatus being produced in the future as the technology develops further.

Mass spectrometry has been increasingly employed for analysis of the same plant species. Fragni *et al*.^43^ investigated forty-one processed tomato samples coming from three different countries by Inductively Coupled Plasma orthogonal acceleration Time-of-Flight Mass Spectrometer (ICP-oa-TOF-MS), exploiting the characteristic of simultaneity of the measurements that is distinctive of this type of instrument. But the objects detected with this method were only metallic elements in plants. Kim *et al*. conducted direct analysis in real time–time of flight–mass spectrometry (DART–TOF–MS) fingerprinting on Dang-gui from Korean and Chinese markets for discriminating geographical origin of raw herbal medicine^44^. Each sample was ground to fine powder and passed through a sieve tube prior to analysis. However, our experiments were done *in situ* of the samples.

## Conclusion

Conclusively, DAPCI is a “green” analytical tool requiring no toxic chemical reagent, with high sensitivity for detection of trace analytes in complex matrices and with high throughput due to no need of any sample pretreatment. As the sheath gas is not required in DAPCI-MS, it is of convenience to implement DAPCI-MS for *in situ* analysis, especially when a portable mass spectrometer installed with a miniaturized DAPCI source is used. Such a DAPCI-MS-based instrument has long been expected for wide application in plant science research, especially for classification the chemotypes of plants, as well as identification of secondary metabolites when the tandem mass spectrometry capability has been implemented in the instrument. As demonstrated in this work, DAPCI-MS would become a powerful analytical tool in many fields of plant science including identification and/or screening of plant chemotypes at the molecular level, showing promising applications on studies of traditional Chinese medicines and *in situ* analysis of Chinese herbs.

## Materials and Methods

### Materials

Fresh leaves of five chemotypes of *C. camphora* trees including the isoborneol-type, camphora-type, cineole-type, linalool-type and borneol-type were picked up at the same time from trees cultured at a trial field authorized by Camphor Engineering Technology Research Center of China National Forestry Administration, on July 28, 2014. The trees of six or seven years old were selected, with 2–4 m height, 21–26 cm diameter at breast height, and 2–3 m canopy. The chemotypes of each *C. camphora* tree has been identified prior to sample collection by Professor Xiangmei Jiang, the Center Director using a standard GC-MS method and transcriptome analysis^17^,^18^, and their chemotypes were confidently determined. Each chemotype was randomly picked from five trees with identified chemotypes, and each individual tree included five orientations, i.e. the east, south, west, north and middle branches. The branches were inserted into water to remain fresh and analyzed after no more than 24 hr.

### Instruments and Working Conditions

All the experiments were carried out using a linear ion trap mass spectrometer (LTQ-XL, Thermo Scientific San Jose, CA) coupled with a homemade DAPCI source for ion generation. The Xcalibur^®^ software was directly used for instrument control and mass spectral raw data display. The DAPCI ion source was placed 10 mm in front of the MS inlet capillary and set at 45° to the sample surface. The gap distance between the needle tip and the sample surface was set to be 1–3 mm, as shown in Fig. 1.

The DAPCI source and LTQ mass spectrometer were both set to run in the positive ion detection mode. The operational parameters such as the capillary voltage, the temperature of the heated capillary, and the tube lens voltage were set to be 15 V, 150 °C, and 65 V, respectively. A high voltage (+3.5 kV, with a discharge current of no more than 0.1 mA) was directly applied to the needle to generate a corona discharge, which produced plenty of primary radical cations and secondary cluster ions to impact the sample surface for desorption/ionization, resulting in analyte ions created under ambient conditions for post mass analysis. The analyte ions are then transferred into the LTQ mass analyzer for mass analysis via the ion guide system of the instrument. The analytical time for each sample was only 30 s. However, the CID mass spectra were averaged over a recording period of 1.6 min to maximize the signal-to-noise ratio of the CID data, followed by background subtraction. For CID experiments, up to MS^3^, the parent ions were selectively isolated with mass-to-charge window width of 1.5 units, and subjected to CID process by applying the collision energy (CE) of 10–30%.

The third expanded fresh leaves of the upper part of each branch in five chemotypes of *C. camphora* were detected in this experiment. The fresh leaves were torn into two pieces parallel with main veins, and the distance between the tear and the main vein was about 1 cm. The tears were placed under DAPCI ion source, as shown in Fig. 1, with manual injection, and the analysis of every sample was repeated three times.

### Data analysis

#### PCA

PCA was performed using Matlab software (version 7.0, Mathworks, Inc., Natick, MA) to recognize the pattern of the raw mass spectral fingerprints. Each mass spectrum was exported to Microsoft Excel and saved as a file for further utilization. The *m/z* values were treated as independent variables and the relative intensities of the full-scan mass spectra (MS^1^) as the dependent variables in the Excel files. To ensure the quality of the statistical analysis, 25 pieces for each chemotype sample of *C. camphora* were collected, and every piece leaf was sampled for 6 times. Hence, there were a total of 750 mass spectra in the data set, which were assembled into an N × 750 matrix. The matrix could be directly used in Matlab software for PCA analysis, where N represents the total number of *m/z* values collected in the mass spectrum. The principal components for the output were automatically determined by Matlab software with the “princomp” function, and the accuracy for PCA (i.e., the factor indicating the accuracy of PCA results; the smaller the factor, the more reliable the results.) was set at a high level (1 × 10^−8^). The scores and loadings of the first three principal components (PCs) were 3D plotted in Matlab.

#### CA

The same data set used for PCA was directly processed with Matlab software (version 7.0, Mathworks, Inc., Natick, MA) for CA, which calculated the Euclidean distance between samples and conducted hierarchical clustering analysis to recognize the pattern based on the mass spectral raw data.

## Additional Information

**How to cite this article**: Guo, X. *et al*. Molecular differentiation of five *Cinnamomum camphora* chemotypes using desorption atmospheric pressure chemical ionization mass spectrometry of raw leaves. *Sci. Rep.* **7**, 46579; doi: 10.1038/srep46579 (2017).

**Publisher's note:** Springer Nature remains neutral with regard to jurisdictional claims in published maps and institutional affiliations.

## Supplementary Material

Supporting Information
