# Supplementary material for: Molecular differentiation of five Cinnamomum camphora chemotypes using desorption atmospheric pressure chemical ionization mass spectrometry of raw leaves
Source: Sci Rep. 2017 Apr 20;7:46579. doi: 10.1038/srep46579 (PMC5397862; doi:10.1038/srep46579)
Supplement: Supporting Information [file srep46579-s1.doc]

**Molecular differentiation of five *Cinnamomum camphora* chemotypes using desorption atmospheric pressure chemical ionization mass spectrometry of raw leaves**

Xiali Guo ^1, 2^, Meng Cui ^1, 2^, Min Deng ^1, 2^, Xingxing Liu ^1, 2^, Xueyong Huang ^1, 2^, Xinglei Zhang ^3^, Liping Luo ^1, 2*^

^1^ School of Life Sciences, Nanchang University, Nanchang, Jiangxi 330031, China

^2^ State Key Laboratory of Food Science and Technology, Nanchang University, Nanchang, Jiangxi 330031, China

^3^ Jiangxi Key Laboratory for Mass Spectrometry and Instrumentation, East China Institute of Technology, Nanchang, Jiangxi 330013, China

*Correspondence and requests for materials should be addressed to Liping Luo. (e-mail: lluo2@126.com).

**Supporting Information**

**Figure S1 CID mass spectral data of protonated Pinene (*m/z* 137)**

**Figure S2 CID mass spectral data of protonated Camphora (*m/z* 153)**

**Figure S3 CID mass spectral data of protonated Linalool (*m/z* 155)**

**Figure S4 CID mass spectral data of protonated Cineole (*m/z* 155)**

**Figure S5 CID mass spectral data of protonated Borneol (*m/z* 155)**
